# Diagnosis of covert coarctation of the aorta in adolescents

**DOI:** 10.3389/fped.2023.1101607

**Published:** 2023-03-21

**Authors:** Luyao Wei, Shijun Hu, Xueyang Gong, Yiliya Ahemaiti, Tianli Zhao

**Affiliations:** Department of Cardiovascular Surgery, The Second Xiangya Hospital, Central South University, Changsha, China

**Keywords:** coarctation of the aorta, underdiagnosis, hypertension, echocardiogram, computed tomography

## Abstract

**Objectives:**

By reviewing the diagnostic process for adolescents with coarctation of the aorta (CoA) in our institution, we analyzed the reasons for delayed diagnosis of CoA. We also proposed a diagnostic protocol to improve the detection rate of CoA.

**Methods:**

In this retrospective study, we included 48 patients aged 12–18 years who were diagnosed with CoA in our hospital from January 2000 to November 2022. Clinical data from involved cases in local hospitals and our institution were collected.

**Results:**

All patients had blood pressure (BP) measurements in upper and lower extremities in our institution. They all had hypertension, 29 (60.4%) of whom had known histories of the same. BP in the upper limbs of 47 (97.9%) patients was ≥20 mmHg higher than that in the lower limbs, and BP in the upper limb of 1 (2.1%) patient was greater than 0 and less than 20 mmHg than that in the lower limb. Echocardiography (ECHO) was performed in all patients, computed tomography (CT) or magnetic resonance imaging (MRI) was performed in 44 patients (91.7%). There were 38 (79.2%) patients who visited local hospitals. Among them, a total of 20 (52.6%) patients had their right upper extremity BP measured, 18 (47.4%) only had their left upper extremity BP measured, and 16 (42.1%) had their lower extremity BP measured. ECHO was performed in 27 (56.2%) patients and CT/MRI was performed in 18 (37.5%) patients. The detection rate for CT/MRI was 100%, and those of ECHO were 72.9% and 18.5% at our institution and a local hospital, respectively. Forty-eight (100%) and 23 (60.5%) patients were detected in our institution and local hospitals (*P* < 0.0001).

**Conclusion:**

We recommend measuring BP in the bilateral upper extremities. Measurement of BP in the lower extremities is recommended if hypertension is diagnosed. MRI/CT is recommended when BP in the upper extremity is greater than that in the lower extremity.

## Introduction

Coarctation of the aorta (CoA) occurs in 0.3 of every 1,000 live births (1) and makes up 5%–8% of all congenital heart diseases (2). It is likely to represent a spectrum of aortic narrowing from a discrete entity to tubular hypoplasia, with many variations seen between the two extremes (3). However, CoA usually manifests as a discrete constriction of the aortic isthmus (4). CoA is also considered as an arteriopathy, given the abnormal histology of the arterial wall close to the site of coarctation and its association with long-term cardiovascular pathology (5). It is the fifth most common cardiovascular anomaly, requiring surgical intervention in infants and children (6).

According to the location of CoA, it is usually divided into the following three types: (i) paraductus, the coarctation is located at the attachment of the ductus arteriosus; (ii) postductus, the constriction is located distal to the opening of the ductus arteriosus; (iii) preductus, where the constriction segment is proximal to the opening of the ductus arteriosus or in the aortic arch.

The main pathophysiological change of CoA is hypertension proximal to coarctation, and the postulated mechanisms of hypertension are mechanical obstruction and renin-angiotensin-mediated humoral mechanisms (2). The increased afterload of the left ventricle will cause left ventricular hypertrophy. Because the cerebral blood vessels are in a state of hypertension for a long time, it can lead to cerebral vascular sclerosis. These conditions improve with the subsequent development of collateral circulation.

The presentations of CoA depend on the severity of narrowing, the relationship to arch vessels, the complexity of comorbidities, and adequacy of collateral vessel formation (7). Therefore, severe cases usually present and are diagnosed during the neonatal period. CoA detected during adolescence and adulthood is often accompanied only by upper extremity hypertension. Some patients can remain asymptomatic until complications of hypertension develop later in life (8).

According to the *2020 ESC Guidelines for the management of adult congenital heart disease* (9), upper extremity BP greater than or equal to 20mmHg lower extremity BP indicates significant CoA. Weak or absent pulses in the lower extremities or radiofemoral pulse delay also indicate significant CoA. The gold standard for the evaluation of significant CoA is cardiac catheterization, which measures proximal and distal BP at the coarctation site. A peak-to-peak gradient across the coarctation site of ≥20 mmHg indicates significant CoA. However, studies (10) had shown that the severity of CoA cannot be accurately assessed by non-invasive measurements of upper and lower limbs BP alone.

Previous studies have suggested that CoA can be safely repaired until early adolescence, preferably before 5 years of age (11–13). As the disease progresses, various vascular complications can occur, such as heart failure, coronary disease, aortic dissection and rupture, hemorrhagic cerebrovascular accident, or bacterial endocarditis. Without correction, the mean life expectancy of patients with CoA is around 35 years, and 90% die of vascular complications before the age of 50 (14), making early diagnosis and treatment of this condition very important.

According to the *Practice Guideline for Screening and Management of High Blood Pressure in Children and Adolescents* (15), the secondary causes of hypertension in adolescence are mainly renal parenchyma and renal vascular diseases, followed by cardiac diseases including CoA and endocrine diseases including pheochromocytoma. At present, there are still many CoA patients who are diagnosed in adolescence and adulthood need surgical treatment (16, 17). Therefore, we suspect that CoA is probably a major cause of hypertension in adolescence, because of the current situation of low diagnostic accuracy.

Therefore, the purpose of this study was to analyze the reasons for delayed diagnosis by reviewing the diagnostic procedures for adolescent patients with CoA in our institution. We also proposed a diagnostic protocol for CoA to improve its diagnostic rate.

## Patients and methods

This study was approved by the Institutional Review Board of the Second Xiangya Hospital (SXH). Informed consent was obtained from all individuals’ legal guardian included in the study.

### Study subjects

We queried patients who were first diagnosed with CoA at SXH of Central South University (Changsha, China) from January 2000 to November 2022, aged 12–18 years, based on their diagnostic codes. There were 48 patients in total, including 32 males (66.7%) and 16 females (33.3%).

Median age at the time of diagnosis with this disease in our institution was 14 years (range, 12–18 years). Diagnosis included isolated CoA in 21 of 48 patients (43.7%). 27 (56.3%) patients had at least one associated congenital cardiac anomaly; of these, 16 (33.3%) had bicuspid aortic valves. 13 (27.1%) patients had previously received non-CoA surgical treatments. There were 5 (10.4%) patients had previously underwent repair of ventricular septal defect (VSD), 3 (6.2%) patients had closure or ligation of patent ductus arteriosus (PDA), and 1 (2.1%) patient had both VSD repair and ligation of PDA. Associated anomalies and previous surgeries are shown in Table 1. One patient (2.1%) had previously undergone aneurysm clipping for an aneurysmal rupture of the anterior cerebral artery.

**Table 1:** Associated anomalies and previous surgeries.

Variables	*N*	Percent
Median age (range)	14 (12–18)	
Sex (male, *n*)	32	66.7
Weight, kg	55.1 ± 3.30	
Height, m	1.64 ± 0.02	
Body mass index	20.2 ± 0.79	
Symptoms at the time of admission
Hypertension	48	100
Shortness of breath	9	18.7
Dizziness	9	18.7
Chest tightness	8	16.7
Headache	6	12.5
Palpitation	4	8.3
Heart murmur	2	4.2
Lower extremity weakness	2	4.2
Associated anomalies
BAV	16	33.3
AR	6	12.5
PDA	6	12.5
ASD	4	8.3
VSD	3	6.2
PFO	1	2.1
QAV	1	2.1
Previous surgeries
Repair of VSD	5	10.4
Closure or ligation of PDA	3	6.2
Appendectomy	2	4.2
Repair of VSD and ligation of PDA	1	2.1
MVP	1	2.1
Aneurysm clipping of the anterior cerebral artery	1	2.1
Complications of hypertension	1	
ICH	1	3.2

BAV, bicuspid aortic valve; AR, aortic regurgitation; PDA, patent ductus arteriosus; ASD, atrial septal defect; VSD, ventricular septal defect; PFO, patent foramen ovale; QAV, quadricuspid aortic valve; MVP, mitral valve plasty; ICH, intracerebral hemorrhage.

### Data acquisition

A retrospectively review of medical records was performed with regard to symptoms, physical examinations, initial diagnosis, echocardiogram (ECHO), computed tomography (CT) and magnetic resonance imaging (MRI), as well as laboratory examinations of patients in local hospitals and our institution. The process of diagnosing these patients was retrospectively analyzed by examination of their hospital admission data. Diagnostic workflows and characteristics were assessed retrospectively.

Our institutional recommendations for the definition of CoA and indications for surgical repair were as follows: (i) increased non-invasive gradient between upper and lower extremities confirmed with invasive cardiac catheterization (peak-to-peak ≥20 mmHg); (ii) hypertensive patients with ≥50% narrowing relative to the aortic diameter at the diaphragm; (iii) patients with normal BP but peak-to-peak ≥20 mmHg by catheterization; or (iv) patients with normal BP but ≥50% narrowing relative to the aortic diameter at the diaphragm.

BP was measured with mercury sphygmomanometer. Hypertension was defined as systolic BP > 130 mmHg and/or diastolic BP > 80 mmHg on the right arm in accordance with the *Clinical Practice Guideline for Screening and Management of High Blood Pressure in Children and Adolescents* (15). Patients with normal BP were also classified as hypertensives if they were on antihypertensive medication at the time of BP measurement.

### Statistics

Descriptive statistics for categorical variables are reported as frequencies and percentages, and continuous variables as either means ± standard deviations (SDs) or as medians and ranges, as appropriate. We used Fisher’s exact test and the *χ*^2^ test to assess differences between categorical variables. We performed all analyses using SPSS version 26 (IBM Corp, Armonk, NY, United States). All statistical tests were two sided, with the *P*-value set at 0.05 for statistical significance.

## Results

### Diagnostic workflow for CoA

#### Reasons for visit in our institution

**Figure 1:**
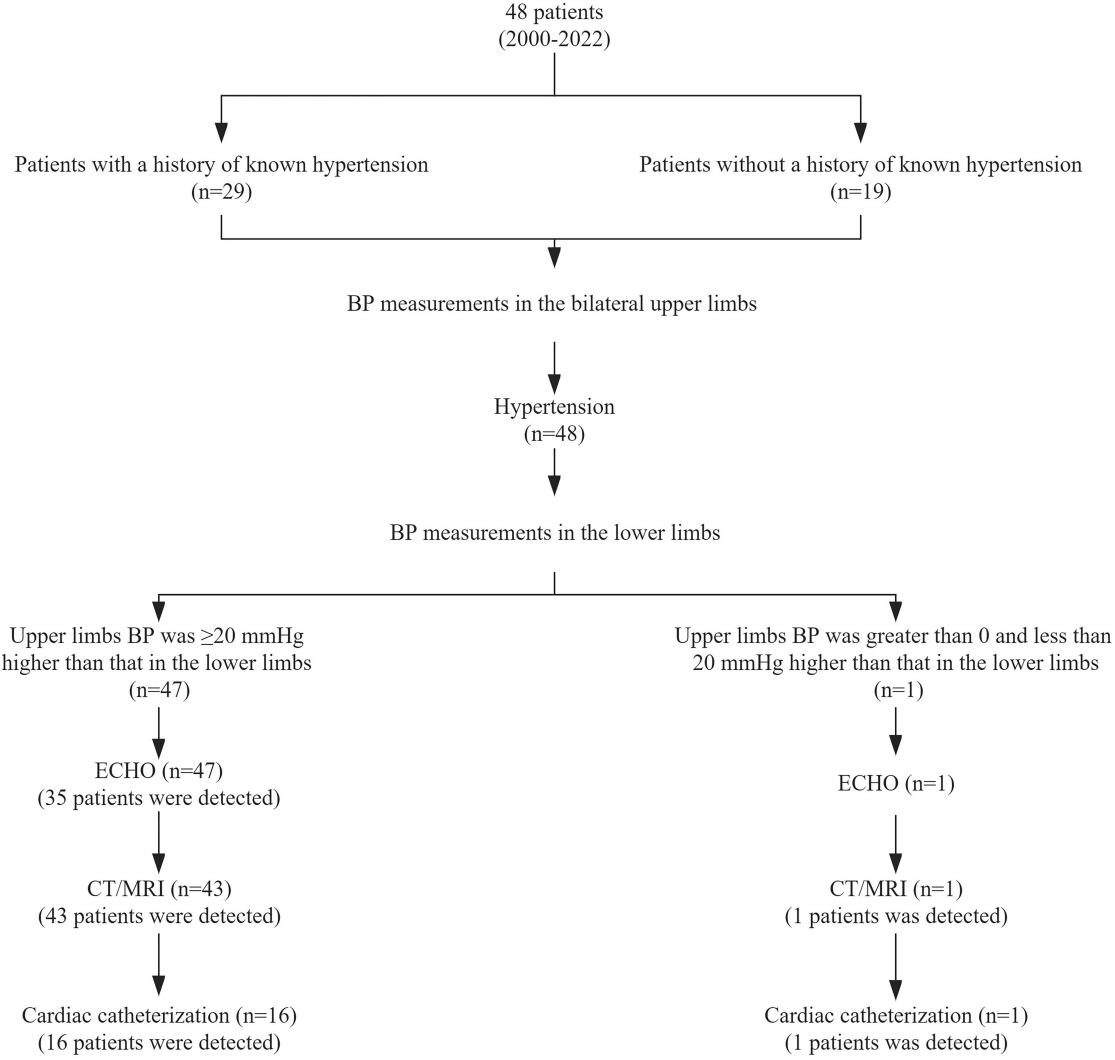
Preoperative diagnostic workflow at our institution, and detection rates of imaging examinations. CoA, coarctation of the aorta; BP, blood pressure; ECHO, echocardiogram; CT, computed tomography; MRI, magnetic resonance imaging.

Twenty-nine (60.4%) patients had previously been diagnosed with hypertension (Figure 1), of whom 14 were admitted to the hospital due to hypertension only; 13 were admitted for hypertension accompanied by headache, dizziness, shortness of breath, lower-extremity weakness, and other symptoms; and 2 was admitted for hypertension accompanied by diastolic murmur in the aortic valve. One patient with known previous hypertension accompanied by headache and dizziness underwent computed-tomography angiography (CTA) of the cranial vessels; bleeding was found in the anterior cerebral artery, and this patient underwent aneurysm clipping. For patients with known previous hypertension, the median time from discovery of hypertension to diagnosis of CoA was 2.5 (range, 0.2–16) months. Nineteen patients had no previous diagnosis of hypertension. Ten of these nineteen were referred to our institution with symptoms such as chest tightness, shortness of breath, headache, dizziness, and palpitations; five of the ten had previously undergone repair of VSD, ligation or occlusion for PDA, or appendectomy. Five of the nineteen aforementioned patients were referred to our institution with systolic murmur at the second and third intercostal spaces at the left sternal border, or continuous mechanical murmur in the second intercostal space at the left sternal border. CoA was found in four patients by routine ECHO assessment at our institution after VSD repair or mitral valve plasty (MVP).

We reviewed the clinical data of patients who had undergone repair of VSD to analyze why CoA was not detected at the same time. Four patients had normal BP in the left upper limb, but BP in the right upper and lower limbs, as well as CT or MRI was not measured. Two patients had normal right upper limb BP, but lower limbs BP and CT or MRI were not measured. One patient had hypertension in the right upper limb. Renal ultrasound and ECHO indicated no abnormalities, and lower extremities BP, CT and MRI were not measured. This patient underwent antihypertensive therapy.

#### BP measurements and imaging examinations performed by local hospitals and their diagnostic rates

**Figure 2:**
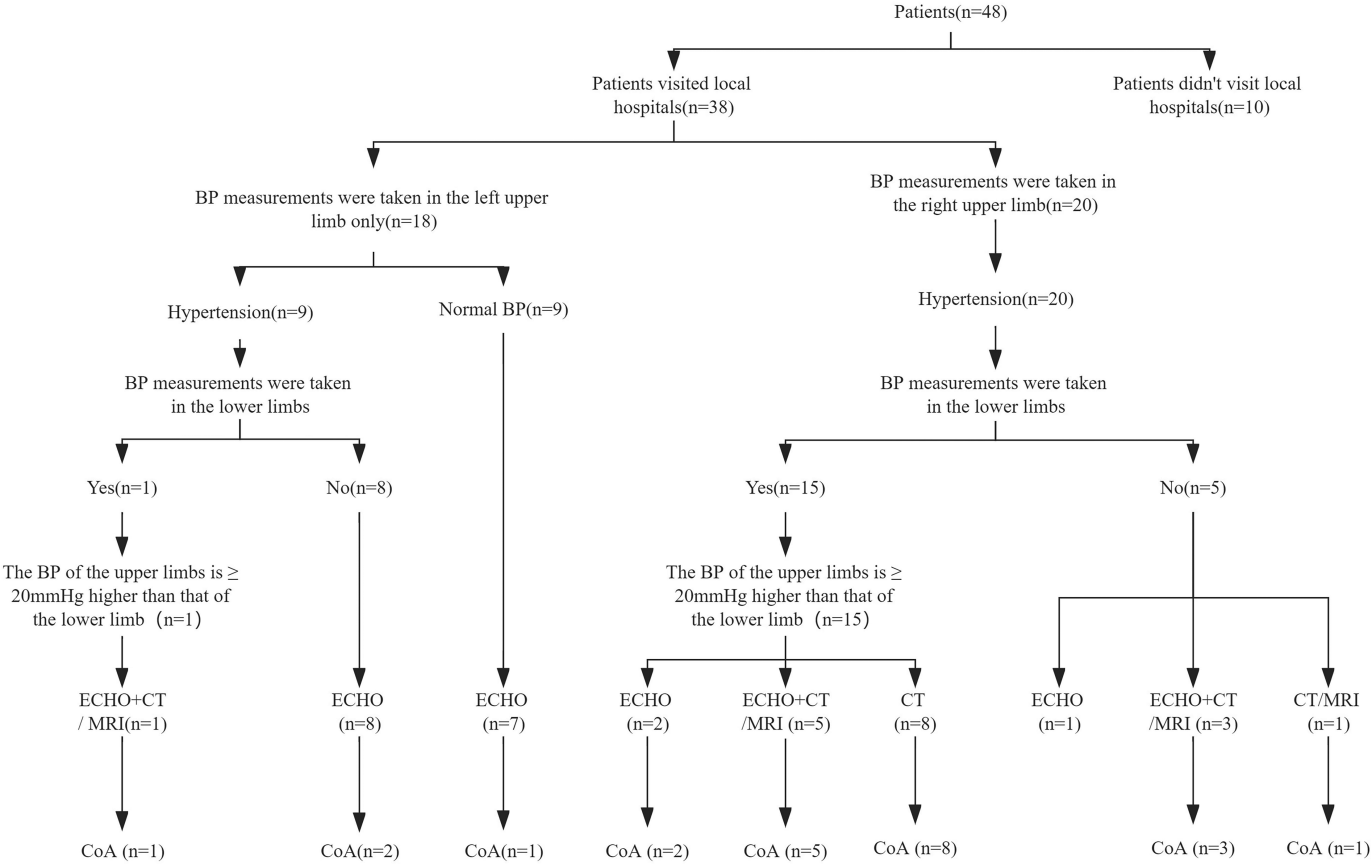
Preoperative diagnostic workflow at local hospitals, and detection rates of imaging examinations at local hospitals.

Of all 48 patients, 38 (79.2%) presented to local hospitals, but 10 (20.8%) were not. Of the patients who presented to the local hospital, 20 had their BP measured in the right upper limb and 18 had their BP measured only in the left upper limb. Among the patients who presented to the local hospital, 20 patients who had their BP measured in the right upper extremity had hypertension, and 18 who had their BP measured only in the left upper extremity had hypertension in 9 patients. Lower extremity BP was measured in 15 patients and 1 patient in the two groups. 18 (47.4%) underwent ECHO alone, 9 (23.7%) underwent CT/MRI, and 9 (23.7%) underwent both types of imaging examination. Of 27 patients who underwent ECHO at the local hospital, 5 were diagnosed with CoA (detection rate, 18.5%). All 18 patients who underwent CT/MRI were diagnosed with CoA (detection rate, 100%). Twenty-three patients were diagnosed with CoA at local hospitals (detection rate, 47.9%). The diagnosis process of patients in local hospitals is shown in Figure 1. Among the remaining 25 patients, 10 patients didn't present to local hospitals, and the remaining 15 patients were not diagnosed accurately in local hospitals, and the causes of hypertension require further examination (Figure 2).

#### BP measurements and imaging examinations performed by our institution and their diagnostic rates

**Figure 3:**
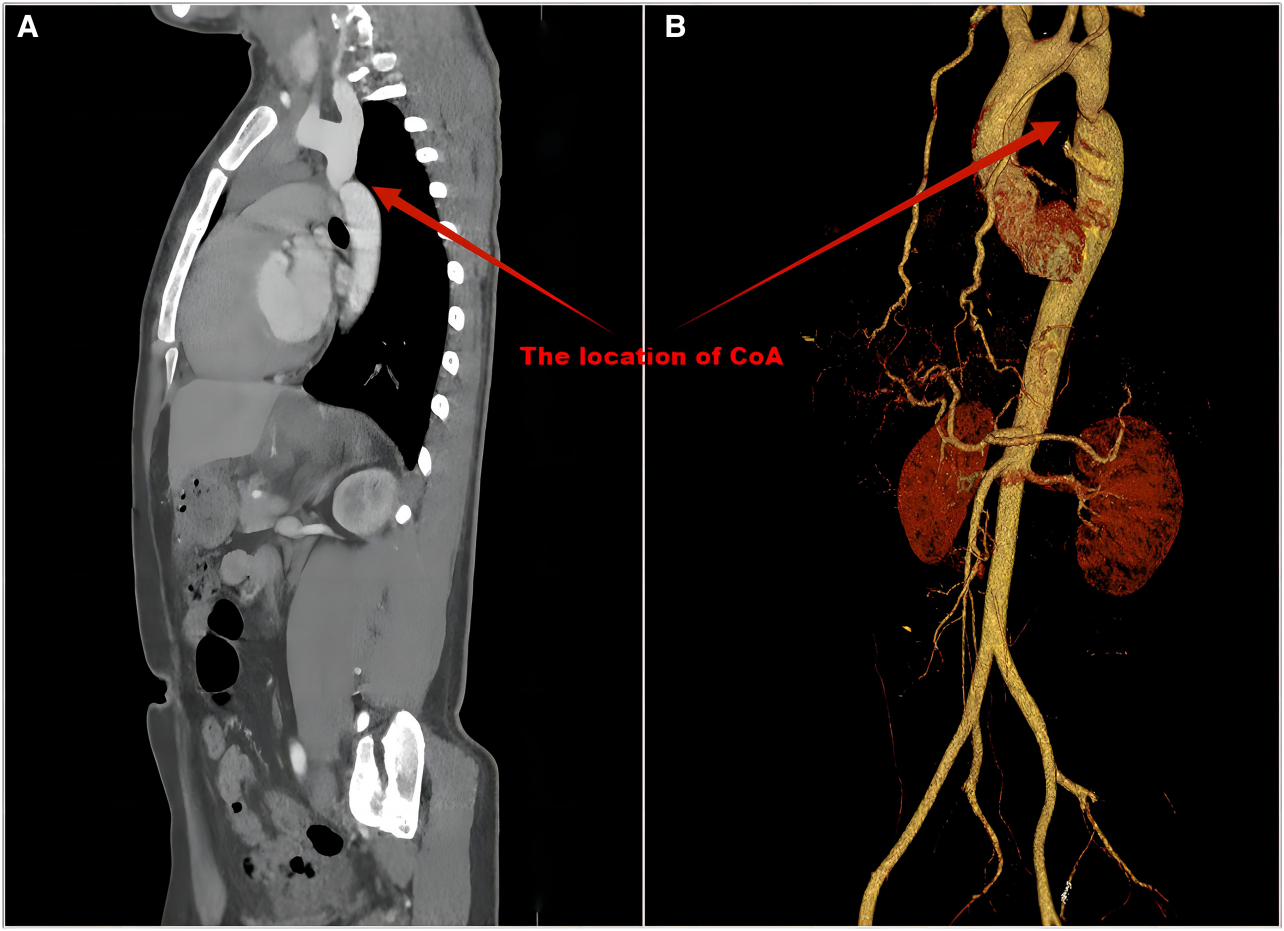
CT (**A**) and 3D reconstruction (**B**) of CoA. The precise location, diameter and length of the narrowing can be shown.

All 48 patients (100%) had BP measurements in upper and lower extremities. Twenty-six of the 48 hypertensive patients received antihypertensive therapy. Three of the 26 patients had normal BP at our institution due to adequate medication. The BP of 47 (97.9%) patients in the upper limb was ≥20 mmHg higher than that in the lower limb, and the BP of 1 patient in the upper limb was greater than 0 and less than 20 mmHg than that in the lower limb. All patients underwent ECHO evaluations, and CoA was detected in 35 (72.9%). CT/MRI was performed in 44 patients and had a CoA detection rate of 100% (Table 2). Seventeen (35.4%) patients underwent cardiac catheterization with a median peak systolic pressure gradient of 34 (range, 20–85) mmHg. CT and 3D reconstruction of CoA are shown in Figure 3. All patients were diagnosed at our institution (Figure 1).

**Table 2:** The number of patients with upper and lower limb BP measurement, ECHO and CT examination.

Variables	Local hospital	Our institution	*P*
BP measurement in right upper extremity (*n*)	20	48	<0.0001
BP measurement in lower extremities (*n*)	16	48	<0.0001
ECHO (*n*)	27	48	<0.0001
MRI/CT (*n*)	18	44	<0.0001
Catheterization	3	17	<0.004
Median peak systolic pressure gradient (range), mm Hg	40 (25–60)	34 (20–85)	

BP, blood pressure; ECHO, echocardiogram; CT, computed tomography; MRI, magnetic resonance imaging.

### Repair techniques

A total of 22 (45.8%) patients underwent open thoracotomy. An extra-anatomic bypass was used in 13 of these patients; surgical modalities included subclavian-artery–descending aortic-bypass graft (4/13), aortic-arch–descending aortic-bypass graft (5/13), and ascending-aorta–descending aortic-bypass graft (4/13). Of the 22 patients, 4 underwent patch aortoplasty, 3 (3/22) underwent end-to-end or extended end-to-end anastomosis, and 2 (2/22) underwent end-to-end anastomosis and subclavian-artery–descending aortic-bypass graft.

A total of 17 (35.4%) patients underwent endovascular (EV) interventions, all of which included an EV stent graft and balloon dilation. Patients received Cheatham-Platinum (CP) stents and balloon-in-balloons (BIBs) developed by NuMED (Orlando, FL, United States). A total of nine (18.8%) patients did not undergo surgical treatment at our hospital.

## Discussion

In the current era of increasingly sophisticated imaging techniques and increasing number of nonthoracic surgical interventions, we sought to assess the process of diagnosing CoA in patients aged 12–18 years at our institution.

Even though most cases of CoA are detected in infancy and childhood, CoA can remain undiagnosed for many years (11, 18). Hypertension is a common symptom of CoA (19), but it is one of the most common symptoms of a wide variety of diseases (20). For adolescents aged 12–18 years, the major etiologies of secondary hypertension include renal diseases and renovascular disease. Uncommon causes include CoA and endocrine causes of hypertension, such as primary aldosteronism (PA), pheochromocytoma, and Cushing’s syndrome (15, 21). However, it is reported in the literature that the overall incidence of renovascular disease is 6.69 per 100,000 person-years, and its incidence is 0.69 in patients aged ≤18 years (22). One study indicated that the prevalence of congenital heart disease is 11.89 cases per 1,000 children (23). Therefore, it could be speculated that CoA is probably a major cause of secondary hypertension and that a diagnosis of CoA in the above-mentioned patients might be missed. CoA should also be prioritized when determining the etiology of hypertension in adolescents.

CoA requires early correction to avoid severe hypertension and vascular complications (12). If not diagnosed early, it often presents with uncontrolled hypertension or hypertension-related complications in adolescence and adulthood (24). For example, a patient with CoA and hypertension in our study developed a ruptured anterior cerebral aneurysm due to the delay in diagnosis. However, according to relevant studies, treatment of CoA in adolescents and adults is common in developing countries due to lack of early detection (25). The patients who did not undergo surgery would suffer a further increase in the degree of obstruction, gradual increases in BP and gradual deterioration of collateral circulation. Operated CoA in adulthood tends to have a poorer prognosis than in childhood and adolescence due to complications of CoA (11). Early diagnosis and treatment of CoA are therefore vital.

Diagnosing hypertension in adolescents is difficult for various reasons, some of which are as follows. Cut-off values for normal BP and hypertension vary by gender, age, and weight in adolescents according to the *2017 Clinical Practice Guidelines for the Screening and Management of Hypertension in Children and Adolescents* (15). Clinician awareness that non-obese adolescents might be diagnosed with hypertension is not yet high (26). Diagnosis of hypertension requires repeated measurement of BP and bilateral BP (15). Of the 18 patients in the present study who had only left arm BP measured at local hospitals, 9 had hypertension, and only four patients were diagnosed with CoA; of the 20 patients who had BP measured in the right arm, all had hypertension, and 19 were diagnosed with CoA. Left upper limb BP will decrease if the CoA is located proximal to the left subclavian artery. Therefore, BP measurements of bilateral upper limbs in the primary hospital are recommended. BP measurement tools are inherently variable (27). Therefore, at present, hypertension in adolescents is highly underdiagnosed. According to surveys, although prevalence rates of hypertension are on the rise in developing countries, there have been no improvements in awareness in these locations, in contrast with developed countries (28, 29).

Normally, BP is 10–20 mmHg higher in the legs than in the arms. Patients with CoA tend to have elevated BP in the upper extremities and decreased BP in the lower ones. BP in the arms is generally ≥20 mmHg higher than in the legs. Of the 23 patients who had the diagnosis of CoA at local hospitals, 16 had their lower extremity BP measured, and seven did not. However, as the disease progresses, collateral circulation gradually develops, and the BP differential between the upper and lower extremities can decrease (16). In our study, BP in the upper limbs of 47 (97.9%) patients was ≥20 mmHg higher than that in the lower limbs, and BP in the upper limb of 1 (2.1%) patient was greater than 0 and less than 20 mmHg higher than that in the lower limb. Therefore, measurement of BP in the lower extremities is recommended when adolescents are diagnosed with hypertension, and CoA needs to be considered if the upper extremity BP is higher than the lower extremity.

The main causes of hypertension in adolescents are kidney disease and essential hypertension (30). Considering that all patients with CoA had hypertension in this study, and considering the high incidence of CoA, we speculate that this condition is probably a major cause of hypertension in adolescents and young adults. It is very likely that many other patients with CoA have not yet been diagnosed.

The disease detection rate of ECHO is lower than that of CT/MRI because physicians tend to ignore the condition of the aorta (31, 32). Recently, CT/MRI has become a principal imaging modality for the evaluation of cardiac and thoracic vascular anomalies due to its short acquisition time and high spatial resolution (33, 34). In our study, among the patients diagnosed with CoA in the local hospital, 18 patients were diagnosed by CT or MRI, and 5 patients were detected by ECHO. The detection rate for ECHO in our institution was 72.9%, and the CoA diagnosis rate for CT/MRI was 100%. However, CT must be replaced by ECHO or MRI in certain patients, such as pregnant women, due to radiation exposure from CT. Note that the detection rate of ECHO depends on the ability of the diagnosing physician. The diagnostic accuracy of ECHO in our institution and local hospitals is not excellent. ECHO is sometimes a priority because MRI is not widely distributed among hospitals and is expensive in China. Moreover, the accuracy rate of ECHO must be improved.

Ten patients did not visit local hospitals, mainly because of the uneven distribution of medical resources between primary and tertiary hospitals in developing countries and the limited medical technology in primary hospitals (35). Patients tend to refer to large hospitals, which also leads to overcrowding of large hospitals and prolonged diagnosis time.

**Figure 4:**
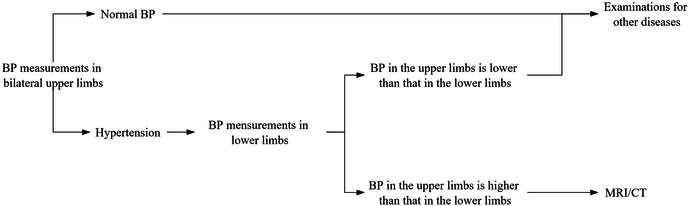
Diagnosis protocol for CoA as per our recommendations.

Given the importance of the timing of diagnosis and the diagnostic process in adolescents with CoA in this manuscript, we have presented a diagnostic protocol (Figure 4). First, bilateral upper BP measurements are recommended in patients at their clinical visit. Second, BP should be measured in lower extremities if upper extremity BP is high (36, 37). Finally, MRI/CT is recommended if the BP in upper limbs is greater than 0mmHg higher than that in lower limbs. We recommended that an ECHO examination is needed if the patient exhibits comorbidities.

### Limitations

The relatively small sample size of this study results in some uncertainty as to the reproducibility of our suggestions. This study is also a single-center study and thus cannot represent the conditions of whole of China or even the developing countries.

### Conclusion

We recommend bilateral upper BP measurements in patients at their clinical visit. If hypertension is diagnosed, we recommend measuring BP in the lower extremities. MRI/CT is recommended when BP in the upper extremity is greater than that in the lower extremity.

## Data Availability

The original contributions presented in the study are included in the article/Supplementary Material, further inquiries can be directed to the corresponding author.

## References

[1] Arya B Maskatia SA. Coarctation of the aorta: prenatal assessment, postnatal management and neonatal outcomes. Semin Perinatol. (2022) 46(4):151584. 10.1016/j.semperi.2022.151584 35422354

[2] Rao PS. Coarctation of the aorta. Semin Nephrol. (1995) 15(2):87–105. PMID: . 7777727

[3] Suradi H Hijazi ZM. Current management of coarctation of the aorta. Glob Cardiol Sci Pract. (2015) 2015(4):44. 10.5339/gcsp.2015.44 26779519 PMC4710863

[4] Kenny D Hijazi ZM. Coarctation of the aorta: from fetal life to adulthood. Cardiol J. (2011) 18(5):487–95. 10.5603/cj.2011.0003 21947983

[5] Dijkema EJ Leiner T Grotenhuis HB. Diagnosis, imaging and clinical management of aortic coarctation. Heart. (2017) 103(15):1148–55. 10.1136/heartjnl-2017-311173 28377475

[6] Brown JW Ruzmetov M Hoyer MH Rodefeld MD Turrentine MW. Recurrent coarctation: is surgical repair of recurrent coarctation of the aorta safe and effective? Ann Thorac Surg. (2009) 88(6):1923–30; discussion 30-1. 10.1016/j.athoracsur.2009.07.024 19932264

[7] Nance JW Ringel RE Fishman EK. Coarctation of the aorta in adolescents and adults: a review of clinical features and CT imaging. J Cardiovasc Comput Tomogr. (2016) 10(1):1–12. 10.1016/j.jcct.2015.11.002 26639936

[8] Qin K Yang J Tang M Iroegbu CD Hu S Fan C. Delayed therapy of descending aortic coarctation results in anterior cerebral rupture: a case report. Front Pediatr. (2021) 9:654705. 10.3389/fped.2021.654705 34671581 PMC8522551

[9] Baumgartner H De Backer J Babu-Narayan SV Budts W Chessa M Diller GP 2020 ESC guidelines for the management of adult congenital heart disease. Eur Heart J. (2021) 42(6):563–645. 10.1093/eurheartj/ehaa554 32860028

[10] Astengo M Berntsson C Johnsson AA Eriksson P Dellbrg M. Ability of noninvasive criteria to predict hemodynamically significant aortic obstruction in adults with coarctation of the aorta. Congenit Heart Dis. (2017) 12(2):174–80. 10.1111/chd.12424 27779371

[11] Lawrie GM DeBakey ME Morris GC Jr., Crawford ES Wagner WF Glaeser DH. Late repair of coarctation of the descending thoracic aorta in 190 patients. Results up to 30 years after operation. Arch Surg. (1981) 116(12):1557–60. 10.1001/archsurg.1981.01380240041006 6459070

[12] Liberthson RR Pennington DG Jacobs ML Daggett WM. Coarctation of the aorta: review of 234 patients and clarification of management problems. Am J Cardiol. (1979) 43(4):835–40. 10.1016/0002-9149(79)90086-9 425922

[13] Hubbell MM Jr O'Brien RG Krovetz LJ Mauck HP Tompkins DG. Status of patients 5 or more years after correction of coarctation of the aorta over age 1 year. Circulation. (1979) 60(1):74–80. 10.1161/01.cir.60.1.74 445735

[14] Campbell M. Natural history of coarctation of the aorta. Br Heart J. (1970) 32(5):633–40. 10.1136/hrt.32.5.633 5470045 PMC487385

[15] Flynn JT Kaelber DC Baker-Smith CM Blowey D Carroll AE Daniels SR Clinical practice guideline for screening and management of high blood pressure in children and adolescents. Pediatrics. (2017) 140(3):e20171904. 10.1542/peds.2017-1904 28827377

[16] Asad I Lee MS Banihani R Wong PD Etoom Y. Coarctation of the aorta in adolescence: significance of detailed cardiac examination in pediatric hypertension. Pediatr Emerg Care. (2021) 37(12):e1724–e5. 10.1097/pec.0000000000001834 30973498

[17] Yin K Zhang Z Lin Y Guo C Sun Y Tian Z Surgical management of aortic coarctation in adolescents and adults. Interact Cardiovasc Thorac Surg. (2017) 24(3):430–5. 10.1093/icvts/ivw353 28011739

[18] Jurcut R Daraban AM Lorber A Deleanu D Amzulescu MS Zara C Coarctation of the aorta in adults: what is the best treatment? Case report and literature review. J Med Life. (2011) 4(2):189–95. PMID: . 21776305 PMC3124275

[19] Rao PS. Coarctation of the aorta. Curr Cardiol Rep. (2005) 7(6):425–34. 10.1007/s11886-005-0060-0 16256011

[20] Siddiqui S Malatesta-Muncher R. Hypertension in children and adolescents: a review of recent guidelines. Pediatr Ann. (2020) 49(6):e250–e7. 10.3928/19382359-20200513-01 32520365

[21] de Freminville JB Amar L. How to explore an endocrine cause of hypertension. J Clin Med. (2022) 11(2):420. 10.3390/jcm11020420 35054115 PMC8780426

[22] Fang CC Chen WJ Peng CL Chen PC Chien KL Tsai TJ Renovascular disease in Taiwan: a long-term nationwide population study. Int J Cardiol. (2013) 168(1):541–2. 10.1016/j.ijcard.2013.01.236 23523252

[23] Marelli AJ Mackie AS Ionescu-Ittu R Rahme E Pilote L. Congenital heart disease in the general population: changing prevalence and age distribution. Circulation. (2007) 115(2):163–72. 10.1161/circulationaha.106.627224 17210844

[24] Wells WJ Prendergast TW Berdjis F Brandl D Lange PE Hetzer R Repair of coarctation of the aorta in adults: the fate of systolic hypertension. Ann Thorac Surg. (1996) 61(4):1168–71. 10.1016/0003-4975(96)00008-2 8607677

[25] Rajbanshi BG Joshi D Pradhan S Gautam NC Timala R Shakya U Primary surgical repair of coarctation of the aorta in adolescents and adults: intermediate results and consequences of hypertension. Eur J Cardiothorac Surg. (2019) 55(2):323–30. 10.1093/ejcts/ezy228 29933438

[26] The fourth report on the diagnosis, evaluation, and treatment of high blood pressure in children and adolescents. Pediatrics. (2004) 114(2 Suppl 4th Report):555–76. PMID: . 15286277

[27] Benson LJ Cohn R Kaelber DC. The association of continuity of care on the diagnosis of hypertension in children and adolescents. J Child Health Care. (2009) 13(4):361–9. 10.1177/1367493509344680 19833670

[28] Ibrahim MM Damasceno A. Hypertension in developing countries. Lancet. (2012) 380(9841):611–9. 10.1016/s0140-6736(12)60861-7 22883510

[29] Tibazarwa KB Damasceno AA. Hypertension in developing countries. Can J Cardiol. (2014) 30(5):527–33. 10.1016/j.cjca.2014.02.020 24786443

[30] Sinaiko AR. Hypertension in children. N Engl J Med. (1996) 335(26):1968–73. 10.1056/nejm199612263352607 8960478

[31] Türkvatan A Akdur PO Olçer T Cumhur T. Coarctation of the aorta in adults: preoperative evaluation with multidetector CT angiography. Diagn Interv Radiol. (2009) 15(4):269–74. 10.4261/1305-3825.Dir.2434-08.1 19847770

[32] Darabian S Zeb I Rezaeian P Razipour A Budoff M. Use of noninvasive imaging in the evaluation of coarctation of aorta. J Comput Assist Tomogr. (2013) 37(1):75–8. 10.1097/RCT.0b013e3182739f81 23321836

[33] Budoff MJ Shittu A Roy S. Use of cardiovascular computed tomography in the diagnosis and management of coarctation of the aorta. J Thorac Cardiovasc Surg. (2013) 146(1):229–32. 10.1016/j.jtcvs.2013.01.024 23395099

[34] Secchi F Iozzelli A Papini GD Aliprandi A Di Leo G Sardanelli F. MR Imaging of aortic coarctation. Radiol Med. (2009) 114(4):524–37. 10.1007/s11547-009-0386-6 19444591

[35] Yin T Liu Z Xu Y. Analysis of crisis management of medical disputes in China and Australia: a narrative review article. Iran J Public Health. (2019) 48(12):2116–23. PMID: . 31993379 PMC6974845

[36] Verberk WJ Kessels AG Thien T. Blood pressure measurement method and inter-arm differences: a meta-analysis. Am J Hypertens. (2011) 24(11):1201–8. 10.1038/ajh.2011.125 21776035

[37] Agasthi P Pujari SH Tseng A Graziano JN Marcotte F Majdalany D Management of adults with coarctation of aorta. World J Cardiol. (2020) 12(5):167–91. 10.4330/wjc.v12.i5.167 32547712 PMC7284000

